# Ethyl 2-{*N*-[*N*-(4-chloro-6-methoxy­pyrimidin-2-yl)carbamo­yl]sulfamo­yl}benzoate

**DOI:** 10.1107/S1600536808017029

**Published:** 2008-06-13

**Authors:** Chui Lu, Fang-Shi Li, Da-Sheng Yu, Wei Yao, Yin-Hong Liu

**Affiliations:** a Department of Applied Chemistry, College of Science, Nanjing University of Technolgy, No. 5 Xinmofan Road, Nanjing 210009, People’s Republic of China

## Abstract

The asymmetric unit of the title compound, C_15_H_15_ClN_4_O_6_S, contains two independent mol­ecules, in which the pyrimidine and benzene rings are oriented at dihedral angles of 75.21 (3) and 86.00 (3)°. Intra­molecular N—H⋯N and C—H⋯O hydrogen bonds result in the formation of two five- and two six-membered rings. The six-membered rings have flattened-boat conformations, while the five-membered rings adopt envelope conformations. In the crystal structure, inter­molecular N—H⋯O hydrogen bonds link the mol­ecules.

## Related literature

For related literature, see: Zhao *et al.* (2006 ▶); Li & Liu (1995 ▶). For bond-length data, see: Allen *et al.* (1987 ▶). For ring puckering parameters, see: Cremer & Pople (1975 ▶).
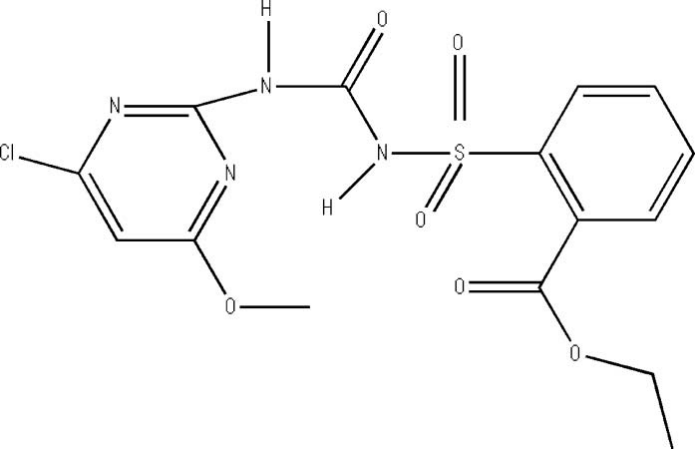


## Experimental

### 

#### Crystal data

C_15_H_15_ClN_4_O_6_S
*M* *_r_* = 414.83
Triclinic, 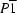

*a* = 7.8210 (16) Å
*b* = 12.310 (3) Å
*c* = 20.200 (4) Å
α = 94.97 (3)°
β = 97.58 (3)°
γ = 93.76 (3)°
*V* = 1914.7 (7) Å^3^
*Z* = 4
Mo *K*α radiation
μ = 0.35 mm^−1^
*T* = 298 (2) K
0.40 × 0.30 × 0.10 mm

#### Data collection

Enraf–Nonius CAD-4 diffractometer
Absorption correction: ψ scan (North *et al.*, 1968 ▶) *T*_min_ = 0.873, *T*_max_ = 0.966
7430 measured reflections
6882 independent reflections
3809 reflections with *I* > 2σ(*I*)
*R*_int_ = 0.069
3 standard reflections frequency: 120 min intensity decay: none

#### Refinement

*R*[*F* ^2^ > 2σ(*F* ^2^)] = 0.088
*wR*(*F* ^2^) = 0.209
*S* = 1.07
6882 reflections
451 parameters
H-atom parameters constrained
Δρ_max_ = 0.39 e Å^−3^
Δρ_min_ = −0.92 e Å^−3^

### 

Data collection: *CAD-4 Software* (Enraf–Nonius, 1989 ▶); cell refinement: *CAD-4 Software*; data reduction: *XCAD4* (Harms & Wocadlo, 1995 ▶); program(s) used to solve structure: *SHELXS97* (Sheldrick, 2008 ▶); program(s) used to refine structure: *SHELXL97* (Sheldrick, 2008 ▶); molecular graphics: *PLATON* (Spek, 2003 ▶); software used to prepare material for publication: *SHELXTL* (Sheldrick, 2008 ▶).

## Supplementary Material

Crystal structure: contains datablocks I, global. DOI: 10.1107/S1600536808017029/hk2471sup1.cif

Structure factors: contains datablocks I. DOI: 10.1107/S1600536808017029/hk2471Isup2.hkl

Additional supplementary materials:  crystallographic information; 3D view; checkCIF report

## Figures and Tables

**Table 1:** Hydrogen-bond geometry (Å, °)

*D*—H⋯*A*	*D*—H	H⋯*A*	*D*⋯*A*	*D*—H⋯*A*
N1—H1*A*⋯N3	0.86	1.96	2.648 (6)	136
N2—H2*A*⋯O5^i^	0.86	2.00	2.831 (8)	162
N5—H5*B*⋯N7	0.86	1.96	2.651 (6)	136
N6—H6*B*⋯O2^ii^	0.86	2.14	2.973 (7)	162
C8—H8*A*⋯O4	0.93	2.43	2.821 (6)	105
C21—H21*A*⋯O9	0.93	2.44	2.848 (8)	107

Symmetry codes: (i) 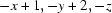; (ii) 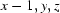.

**Fig. 1.**
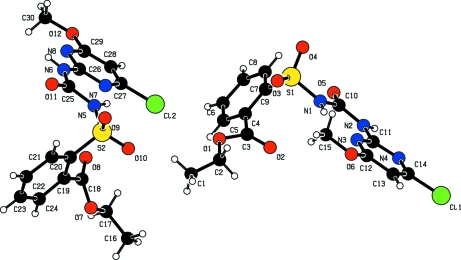
The molecular structure of the title molecule, with the atom-numbering scheme.

**Fig. 2.**
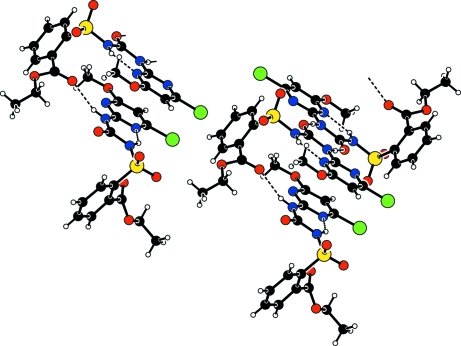
A partial packing diagram of (I). Hydrogen bonds are shown as dashed lines.

## supplementary crystallographic information

### Comment

The title compound, (I), is a highly effective postemergence sulfonylurea herbicide used to control many annual broadleaf weeds in soybean fields (Zhao *et al.*,  2006). As part of our studies in this area, we report herein its crystal structure.

The asymmetric unit of (I) (Fig. 1) contains two independent molecules. The bond lengths (Allen *et al.*,  1987) and angles are within normal ranges. Rings A (C4-C9), B (N3/N4/C11-C14) and C (C19-C24), D (N7/N8/C26-C29) are, of course, planar, and the dihedral angles between them are A/B = 75.21 (3)° and C/D = 86.00 (3)°. The intramolecular N-H···N and C-H···O hydrogen bonds (Table 1) result in the formation of two five- and two six-membered rings: E (N1-N3/C10/C11/H1A), F (S1/O4/C8/C9/H8A), G (N5-N7/C25/C26/H5B) and H (S2/O9/C20/C21/H21A). Rings E and G adopt flattened-boat conformations, having total puckering amplitudes, Q_T_, of 0.139 (3) and 0.117 (3) Å (Cremer & Pople, 1975), while rings F and H have envelope conformations, with O4 and O9 atoms displaced by 0.291 (3) and -0.182 (3) Å from the planes of the other ring atoms, respectively.

In the crystal structure, intermolecular N-H···O hydrogen bonds (Table 1) link the molecules (Fig. 2), in which they may be effective in the stabilization of the structure.

### Experimental

The title compound, (I), was prepared according to the literature method (Li & Liu, 1995). Crystals suitable for X-ray analysis were obtained by dissolving (I) (0.1 g) in acetonitrile (25 ml) and evaporating the solvent slowly at room temperature for about 7 d.

### Refinement

H atoms were positioned geometrically, with N-H = 0.86 Å (for NH) and C-H = 0.93, 0.97 and 0.96 Å for aromatic, methylene and methyl H and constrained to ride on their parent atoms with U_iso_(H) = xU_eq_(C,N), where x = 1.5 for methyl H and x = 1.2 for all other H atoms.

### Crystal data

**Table:**

C_15_H_15_ClN_4_O_6_S	*Z* = 4
*M**_r_* = 414.83	*F*_000_ = 856
Triclinic, *P*1	*D*_x_ = 1.439 Mg m^−^^3^
Hall symbol: -P 1	Mo *K*α radiation λ = 0.71073 Å
*a* = 7.8210 (16) Å	Cell parameters from 25 reflections
*b* = 12.310 (3) Å	θ = 10–13º
*c* = 20.200 (4) Å	µ = 0.35 mm^−^^1^
α = 94.97 (3)º	*T* = 298 (2) K
β = 97.58 (3)º	Block, colorless
γ = 93.76 (3)º	0.40 × 0.30 × 0.10 mm
*V* = 1914.7 (7) Å^3^	

### Data collection

**Table:**

Enraf–Nonius CAD-4 diffractometer	*R*_int_ = 0.069
Radiation source: fine-focus sealed tube	θ_max_ = 25.2º
Monochromator: graphite	θ_min_ = 1.0º
*T* = 298(2) K	*h* = −9→9
ω/2θ scans	*k* = −14→14
Absorption correction: ψ scan(North *et al.*, 1968)	*l* = 0→24
*T*_min_ = 0.873, *T*_max_ = 0.966	3 standard reflections
7430 measured reflections	every 120 min
6882 independent reflections	intensity decay: none
3809 reflections with *I* > 2σ(*I*)	

### Refinement

**Table:**

Refinement on *F*^2^	Secondary atom site location: difference Fourier map
Least-squares matrix: full	Hydrogen site location: inferred from neighbouring sites
*R*[*F*^2^ > 2σ(*F*^2^)] = 0.088	H-atom parameters constrained
*wR*(*F*^2^) = 0.209	*w* = 1/[σ^2^(*F*_o_^2^) + (0.06*P*)^2^ + 3*P*] where *P* = (*F*_o_^2^ + 2*F*_c_^2^)/3
*S* = 1.07	(Δ/σ)_max_ < 0.001
6882 reflections	Δρ_max_ = 0.39 e Å^−^^3^
451 parameters	Δρ_min_ = −0.92 e Å^−^^3^
Primary atom site location: structure-invariant direct methods	Extinction correction: none

### Special details

**Table:**

Geometry. All e.s.d.'s (except the e.s.d. in the dihedral angle between two l.s. planes) are estimated using the full covariance matrix. The cell e.s.d.'s are taken into account individually in the estimation of e.s.d.'s in distances, angles and torsion angles; correlations between e.s.d.'s in cell parameters are only used when they are defined by crystal symmetry. An approximate (isotropic) treatment of cell e.s.d.'s is used for estimating e.s.d.'s involving l.s. planes.
Refinement. Refinement of F^2^ against ALL reflections. The weighted R-factor wR and goodness of fit S are based on F^2^, conventional R-factors R are based on F, with F set to zero for negative F^2^. The threshold expression of F^2^ > 2sigma(F^2^) is used only for calculating R-factors(gt) etc. and is not relevant to the choice of reflections for refinement. R-factors based on F^2^ are statistically about twice as large as those based on F, and R- factors based on ALL data will be even larger.

### Fractional atomic coordinates and isotropic or equivalent isotropic displacement parameters (Å^2^)

**Table:**

	*x*	*y*	*z*	*U*_iso_*/*U*_eq_	
Cl1	0.2819 (3)	1.35508 (17)	0.20491 (14)	0.1456 (9)	
Cl2	−0.2214 (4)	1.36337 (15)	0.18268 (12)	0.1378 (9)	
S1	0.30971 (16)	0.69683 (11)	0.07830 (8)	0.0633 (4)	
S2	−0.4009 (2)	1.21087 (14)	0.41116 (9)	0.0851 (5)	
O1	0.6496 (7)	0.6052 (5)	0.2354 (3)	0.121	
O2	0.5614 (6)	0.7692 (4)	0.2171 (2)	0.1023 (14)	
O3	0.2506 (5)	0.6505 (3)	0.1335 (2)	0.0809 (11)	
O4	0.2122 (5)	0.6758 (3)	0.0137 (2)	0.0790 (11)	
O5	0.4435 (4)	0.8758 (3)	0.00933 (17)	0.0617 (9)	
O6	0.1314 (6)	0.9834 (4)	0.27791 (19)	0.0895 (12)	
O7	0.1026 (6)	1.3566 (4)	0.4533 (2)	0.1042 (14)	
O8	−0.0191 (6)	1.2479 (4)	0.3635 (2)	0.0983 (14)	
O9	−0.5648 (6)	1.1767 (5)	0.4287 (3)	0.1230 (18)	
O10	−0.3643 (6)	1.3245 (4)	0.4050 (2)	0.1082 (15)	
O11	−0.4315 (5)	0.9729 (3)	0.3604 (2)	0.0879 (12)	
O12	−0.1102 (5)	1.0051 (3)	0.0628 (2)	0.087	
N1	0.3246 (5)	0.8291 (3)	0.0994 (2)	0.0621 (11)	
H1A	0.2927	0.8530	0.1368	0.074*	
N2	0.3823 (5)	1.0128 (3)	0.0822 (2)	0.0562 (10)	
H2A	0.4246	1.0587	0.0578	0.067*	
N3	0.2572 (5)	0.9970 (4)	0.1804 (2)	0.0645 (11)	
N4	0.3277 (5)	1.1683 (4)	0.1435 (2)	0.0727 (12)	
N5	−0.3771 (5)	1.1487 (4)	0.3380 (2)	0.0696 (12)	
H5B	−0.3591	1.1887	0.3064	0.084*	
N6	−0.3321 (5)	1.0026 (4)	0.2635 (2)	0.0664 (11)	
H6B	−0.3406	0.9328	0.2543	0.080*	
N7	−0.2656 (6)	1.1691 (4)	0.2210 (2)	0.0780 (13)	
N8	−0.2204 (5)	0.9995 (4)	0.1625 (2)	0.0710 (12)	
C1	0.7097 (10)	0.5750 (6)	0.3555 (4)	0.110	
H1B	0.6547	0.5830	0.3953	0.166*	
H1C	0.8283	0.6036	0.3658	0.166*	
H1D	0.7048	0.4989	0.3396	0.166*	
C2	0.6224 (10)	0.6335 (6)	0.3050 (3)	0.105	
H2B	0.6568	0.7108	0.3163	0.127*	
H2C	0.4993	0.6235	0.3071	0.127*	
C3	0.6109 (8)	0.6821 (5)	0.1952 (4)	0.0849 (19)	
C4	0.6389 (7)	0.6488 (4)	0.1286 (4)	0.0716 (15)	
C5	0.8039 (7)	0.6113 (4)	0.1169 (4)	0.087 (2)	
H5A	0.8878	0.6041	0.1530	0.104*	
C6	0.8363 (9)	0.5873 (5)	0.0558 (6)	0.103 (3)	
H6A	0.9405	0.5580	0.0498	0.123*	
C7	0.7241 (9)	0.6033 (5)	0.0002 (4)	0.095 (2)	
H7A	0.7559	0.5902	−0.0424	0.113*	
C8	0.5624 (7)	0.6392 (4)	0.0077 (4)	0.0794 (17)	
H8A	0.4828	0.6478	−0.0296	0.095*	
C9	0.5227 (6)	0.6617 (4)	0.0718 (3)	0.0610 (13)	
C10	0.3871 (6)	0.9035 (4)	0.0590 (3)	0.0562 (12)	
C11	0.3213 (6)	1.0596 (4)	0.1379 (3)	0.0585 (12)	
C12	0.2003 (7)	1.0443 (5)	0.2328 (3)	0.0688 (15)	
C13	0.2059 (8)	1.1576 (6)	0.2442 (3)	0.0843 (18)	
H13A	0.1685	1.1925	0.2818	0.101*	
C14	0.2698 (8)	1.2139 (5)	0.1968 (3)	0.0809 (17)	
C15	0.1267 (10)	0.8714 (7)	0.2624 (4)	0.113 (2)	
H15A	0.0762	0.8347	0.2960	0.169*	
H15B	0.2422	0.8502	0.2610	0.169*	
H15C	0.0584	0.8515	0.2195	0.169*	
C16	0.2150 (10)	1.5357 (6)	0.4433 (4)	0.119	
H16A	0.2730	1.5823	0.4161	0.178*	
H16B	0.2847	1.5350	0.4861	0.178*	
H16C	0.1053	1.5626	0.4497	0.178*	
C17	0.1872 (10)	1.4226 (6)	0.4092 (4)	0.110	
H17A	0.1149	1.4217	0.3662	0.132*	
H17B	0.2969	1.3947	0.4017	0.132*	
C18	0.0078 (8)	1.2714 (5)	0.4211 (3)	0.0780 (16)	
C19	−0.0646 (8)	1.2030 (5)	0.4724 (3)	0.0746 (15)	
C20	−0.2409 (8)	1.1672 (5)	0.4701 (3)	0.0764 (15)	
C21	−0.2891 (9)	1.1016 (5)	0.5153 (3)	0.0913 (18)	
H21A	−0.4058	1.0800	0.5142	0.110*	
C22	−0.1676 (12)	1.0649 (6)	0.5642 (4)	0.110 (2)	
H22A	−0.2028	1.0171	0.5939	0.132*	
C23	−0.0037 (11)	1.0987 (7)	0.5676 (4)	0.109 (2)	
H23A	0.0758	1.0747	0.6005	0.131*	
C24	0.0571 (10)	1.1704 (6)	0.5229 (4)	0.106 (2)	
H24A	0.1734	1.1950	0.5270	0.127*	
C25	−0.3841 (7)	1.0362 (5)	0.3238 (3)	0.0684 (14)	
C26	−0.2682 (6)	1.0601 (5)	0.2143 (3)	0.0675 (13)	
C27	−0.2115 (9)	1.2230 (6)	0.1711 (4)	0.0914 (18)	
C28	−0.1619 (9)	1.1722 (6)	0.1197 (4)	0.0905 (18)	
H28A	−0.1251	1.2107	0.0859	0.109*	
C29	−0.1637 (7)	1.0629 (5)	0.1155 (3)	0.0747 (15)	
C30	−0.1119 (5)	0.8938 (3)	0.0581 (2)	0.0413 (10)	
H30A	−0.0725	0.8680	0.0170	0.062*	
H30B	−0.2276	0.8629	0.0587	0.062*	
H30C	−0.0369	0.8720	0.0954	0.062*	

### Atomic displacement parameters (Å^2^)

**Table:**

	*U*^11^	*U*^22^	*U*^33^	*U*^12^	*U*^13^	*U*^23^
Cl1	0.163 (2)	0.0865 (13)	0.183 (2)	−0.0031 (13)	0.0484 (17)	−0.0403 (14)
Cl2	0.199 (2)	0.0701 (12)	0.1447 (19)	0.0120 (13)	0.0191 (16)	0.0190 (11)
S1	0.0420 (7)	0.0561 (8)	0.0927 (10)	−0.0030 (6)	0.0109 (7)	0.0143 (7)
S2	0.0714 (10)	0.0853 (12)	0.0964 (12)	0.0066 (8)	0.0153 (9)	−0.0094 (9)
O1	0.121	0.121	0.121	0.009	0.017	0.012
O2	0.131 (4)	0.064 (3)	0.109 (3)	0.008 (3)	0.009 (3)	−0.001 (2)
O3	0.066 (2)	0.070 (2)	0.113 (3)	0.0030 (19)	0.025 (2)	0.030 (2)
O4	0.071 (3)	0.078 (3)	0.085 (3)	−0.009 (2)	0.007 (2)	0.010 (2)
O5	0.058 (2)	0.060 (2)	0.069 (2)	0.0027 (16)	0.0199 (18)	0.0014 (17)
O6	0.092 (3)	0.121 (4)	0.062 (2)	0.027 (3)	0.018 (2)	0.018 (2)
O7	0.100 (3)	0.094 (3)	0.112 (4)	−0.023 (3)	0.021 (3)	−0.013 (3)
O8	0.099 (3)	0.114 (4)	0.077 (3)	−0.015 (3)	0.014 (2)	−0.007 (3)
O9	0.072 (3)	0.151 (5)	0.145 (4)	−0.003 (3)	0.041 (3)	−0.026 (3)
O10	0.109 (4)	0.072 (3)	0.134 (4)	0.019 (3)	−0.010 (3)	−0.019 (3)
O11	0.093 (3)	0.082 (3)	0.088 (3)	−0.017 (2)	0.018 (2)	0.011 (2)
O12	0.087	0.087	0.087	0.007	0.012	0.008
N1	0.050 (2)	0.060 (3)	0.081 (3)	0.013 (2)	0.017 (2)	0.012 (2)
N2	0.045 (2)	0.059 (3)	0.066 (3)	0.0026 (19)	0.0112 (19)	0.010 (2)
N3	0.051 (3)	0.082 (3)	0.061 (3)	0.020 (2)	0.002 (2)	0.009 (2)
N4	0.054 (3)	0.066 (3)	0.093 (3)	−0.002 (2)	0.005 (2)	−0.010 (3)
N5	0.068 (3)	0.072 (3)	0.064 (3)	0.006 (2)	−0.005 (2)	−0.002 (2)
N6	0.067 (3)	0.065 (3)	0.063 (3)	0.000 (2)	−0.005 (2)	0.010 (2)
N7	0.082 (3)	0.078 (3)	0.073 (3)	0.004 (3)	0.002 (2)	0.014 (2)
N8	0.053 (3)	0.088 (3)	0.067 (3)	0.004 (2)	−0.007 (2)	0.005 (2)
C1	0.110	0.110	0.110	0.009	0.015	0.011
C2	0.105	0.105	0.105	0.008	0.015	0.010
C3	0.079 (4)	0.062 (4)	0.109 (5)	0.000 (3)	−0.015 (4)	0.034 (4)
C4	0.051 (3)	0.045 (3)	0.116 (5)	−0.002 (2)	0.002 (3)	0.012 (3)
C5	0.048 (3)	0.047 (3)	0.164 (7)	−0.004 (3)	0.015 (4)	0.001 (4)
C6	0.048 (4)	0.056 (4)	0.205 (9)	0.000 (3)	0.043 (5)	−0.019 (5)
C7	0.071 (4)	0.052 (3)	0.163 (7)	−0.015 (3)	0.057 (5)	−0.021 (4)
C8	0.060 (4)	0.058 (3)	0.120 (5)	−0.009 (3)	0.014 (3)	0.013 (3)
C9	0.049 (3)	0.041 (3)	0.090 (4)	−0.011 (2)	0.010 (3)	0.005 (3)
C10	0.036 (3)	0.056 (3)	0.077 (4)	0.010 (2)	0.005 (2)	0.014 (3)
C11	0.043 (3)	0.066 (3)	0.063 (3)	0.005 (2)	−0.004 (2)	0.004 (3)
C12	0.052 (3)	0.098 (5)	0.054 (3)	0.014 (3)	−0.003 (3)	0.006 (3)
C13	0.067 (4)	0.113 (6)	0.069 (4)	0.015 (4)	0.007 (3)	−0.017 (4)
C14	0.069 (4)	0.079 (4)	0.086 (4)	0.000 (3)	−0.006 (3)	−0.013 (3)
C15	0.126 (6)	0.122 (7)	0.096 (5)	−0.004 (5)	0.026 (4)	0.037 (5)
C16	0.119	0.119	0.119	0.009	0.016	0.011
C17	0.110	0.110	0.110	0.009	0.015	0.011
C18	0.064 (4)	0.082 (4)	0.084 (4)	−0.007 (3)	0.012 (3)	−0.007 (3)
C19	0.078 (4)	0.080 (4)	0.065 (3)	−0.009 (3)	0.017 (3)	0.001 (3)
C20	0.076 (3)	0.075 (4)	0.074 (3)	−0.006 (3)	0.008 (3)	−0.006 (3)
C21	0.094 (4)	0.095 (4)	0.083 (4)	−0.019 (3)	0.025 (3)	0.001 (3)
C22	0.138 (5)	0.110 (5)	0.075 (4)	−0.012 (4)	0.008 (4)	0.006 (3)
C23	0.127 (5)	0.117 (5)	0.079 (4)	0.015 (4)	−0.008 (4)	0.015 (4)
C24	0.094 (4)	0.114 (5)	0.099 (5)	−0.002 (4)	−0.010 (3)	−0.005 (4)
C25	0.056 (3)	0.074 (4)	0.073 (4)	0.002 (3)	−0.002 (3)	0.011 (3)
C26	0.042 (3)	0.091 (4)	0.064 (3)	−0.002 (3)	−0.004 (2)	0.003 (3)
C27	0.088 (4)	0.101 (4)	0.082 (4)	0.012 (3)	−0.004 (3)	0.013 (3)
C28	0.092 (4)	0.086 (4)	0.090 (4)	−0.002 (3)	0.004 (3)	0.017 (3)
C29	0.058 (3)	0.099 (4)	0.063 (3)	0.010 (3)	−0.005 (3)	0.002 (3)
C30	0.030 (2)	0.050 (3)	0.041 (2)	0.0020 (18)	0.0019 (17)	−0.0027 (18)

### Geometric parameters (Å, °)

**Table:**

Cl1—C14	1.727 (7)	Cl2—C27	1.732 (7)
S1—O3	1.413 (4)	S2—O9	1.421 (5)
S1—O4	1.416 (4)	S2—O10	1.429 (5)
S1—N1	1.639 (4)	S2—N5	1.643 (4)
S1—C9	1.767 (5)	S2—C20	1.755 (6)
O1—C3	1.325 (7)	O7—C18	1.314 (7)
O1—C2	1.464 (8)	O7—C17	1.450 (8)
O2—C3	1.230 (8)	O8—C18	1.161 (7)
O5—C10	1.180 (6)	O11—C25	1.195 (6)
O6—C12	1.370 (7)	O12—C29	1.356 (6)
O6—C15	1.384 (8)	O12—C30	1.364 (5)
N1—C10	1.386 (6)	N5—C25	1.385 (7)
N1—H1A	0.8600	N5—H5B	0.8600
N2—C11	1.374 (6)	N6—C25	1.373 (7)
N2—C10	1.391 (6)	N6—C26	1.395 (7)
N2—H2A	0.8600	N6—H6B	0.8600
N3—C12	1.306 (6)	N7—C26	1.335 (7)
N3—C11	1.327 (6)	N7—C27	1.352 (8)
N4—C14	1.319 (7)	N8—C26	1.341 (7)
N4—C11	1.331 (6)	N8—C29	1.380 (7)
C1—C2	1.427 (7)	C16—C17	1.488 (9)
C1—H1B	0.9600	C16—H16A	0.9600
C1—H1C	0.9600	C16—H16B	0.9600
C1—H1D	0.9600	C16—H16C	0.9600
C2—H2B	0.9700	C17—H17A	0.9700
C2—H2C	0.9700	C17—H17B	0.9700
C3—C4	1.422 (9)	C18—C19	1.533 (8)
C4—C9	1.393 (8)	C19—C24	1.404 (9)
C4—C5	1.440 (8)	C19—C20	1.413 (8)
C5—C6	1.305 (10)	C20—C21	1.344 (8)
C5—H5A	0.9300	C21—C22	1.403 (10)
C6—C7	1.369 (10)	C21—H21A	0.9300
C6—H6A	0.9300	C22—C23	1.313 (10)
C7—C8	1.389 (8)	C22—H22A	0.9300
C7—H7A	0.9300	C23—C24	1.420 (10)
C8—C9	1.381 (8)	C23—H23A	0.9300
C8—H8A	0.9300	C24—H24A	0.9300
C12—C13	1.391 (8)	C27—C28	1.282 (9)
C13—C14	1.357 (8)	C28—C29	1.339 (8)
C13—H13A	0.9300	C28—H28A	0.9300
C15—H15A	0.9600	C30—H30A	0.9600
C15—H15B	0.9600	C30—H30B	0.9600
C15—H15C	0.9600	C30—H30C	0.9600
			
O3—S1—O4	120.0 (2)	O9—S2—O10	118.1 (3)
O3—S1—N1	104.5 (2)	O9—S2—N5	109.7 (3)
O4—S1—N1	108.2 (2)	O10—S2—N5	104.9 (3)
O3—S1—C9	109.6 (2)	O9—S2—C20	107.9 (3)
O4—S1—C9	107.6 (3)	O10—S2—C20	109.1 (3)
N1—S1—C9	106.0 (2)	N5—S2—C20	106.5 (2)
C3—O1—C2	113.9 (6)	C18—O7—C17	113.0 (5)
C12—O6—C15	114.6 (5)	C29—O12—C30	121.9 (5)
C10—N1—S1	122.1 (4)	C25—N5—S2	124.5 (4)
C10—N1—H1A	119.0	C25—N5—H5B	117.8
S1—N1—H1A	119.0	S2—N5—H5B	117.8
C11—N2—C10	130.6 (4)	C25—N6—C26	132.3 (5)
C11—N2—H2A	114.7	C25—N6—H6B	113.9
C10—N2—H2A	114.7	C26—N6—H6B	113.9
C12—N3—C11	118.6 (5)	C26—N7—C27	117.3 (5)
C14—N4—C11	115.5 (5)	C26—N8—C29	112.1 (5)
C2—C1—H1B	109.5	C17—C16—H16A	109.5
C2—C1—H1C	109.5	C17—C16—H16B	109.5
H1B—C1—H1C	109.5	H16A—C16—H16B	109.5
C2—C1—H1D	109.5	C17—C16—H16C	109.5
H1B—C1—H1D	109.5	H16A—C16—H16C	109.5
H1C—C1—H1D	109.5	H16B—C16—H16C	109.5
C1—C2—O1	117.7 (6)	O7—C17—C16	106.5 (6)
C1—C2—H2B	107.9	O7—C17—H17A	110.4
O1—C2—H2B	107.9	C16—C17—H17A	110.4
C1—C2—H2C	107.9	O7—C17—H17B	110.4
O1—C2—H2C	107.9	C16—C17—H17B	110.4
H2B—C2—H2C	107.2	H17A—C17—H17B	108.6
O2—C3—O1	120.8 (7)	O8—C18—O7	127.7 (6)
O2—C3—C4	128.4 (6)	O8—C18—C19	123.5 (6)
O1—C3—C4	110.8 (6)	O7—C18—C19	108.8 (5)
C9—C4—C3	123.8 (5)	C24—C19—C20	119.1 (6)
C9—C4—C5	116.4 (6)	C24—C19—C18	116.2 (6)
C3—C4—C5	119.4 (6)	C20—C19—C18	124.6 (6)
C6—C5—C4	120.4 (7)	C21—C20—C19	119.8 (6)
C6—C5—H5A	119.8	C21—C20—S2	118.8 (5)
C4—C5—H5A	119.8	C19—C20—S2	121.3 (5)
C5—C6—C7	123.0 (6)	C20—C21—C22	121.6 (7)
C5—C6—H6A	118.5	C20—C21—H21A	119.2
C7—C6—H6A	118.5	C22—C21—H21A	119.2
C6—C7—C8	119.6 (7)	C23—C22—C21	119.3 (7)
C6—C7—H7A	120.2	C23—C22—H22A	120.3
C8—C7—H7A	120.2	C21—C22—H22A	120.3
C9—C8—C7	118.4 (7)	C22—C23—C24	122.6 (8)
C9—C8—H8A	120.8	C22—C23—H23A	118.7
C7—C8—H8A	120.8	C24—C23—H23A	118.7
C8—C9—C4	122.1 (5)	C19—C24—C23	117.6 (7)
C8—C9—S1	116.4 (4)	C19—C24—H24A	121.2
C4—C9—S1	121.3 (4)	C23—C24—H24A	121.2
O5—C10—N1	122.3 (5)	O11—C25—N6	122.1 (6)
O5—C10—N2	122.7 (5)	O11—C25—N5	123.4 (6)
N1—C10—N2	114.9 (5)	N6—C25—N5	114.4 (5)
N3—C11—N4	124.6 (5)	N7—C26—N8	125.6 (5)
N3—C11—N2	120.2 (5)	N7—C26—N6	118.2 (5)
N4—C11—N2	115.2 (5)	N8—C26—N6	116.1 (5)
N3—C12—O6	120.8 (6)	C28—C27—N7	121.7 (7)
N3—C12—C13	121.3 (6)	C28—C27—Cl2	124.5 (6)
O6—C12—C13	117.9 (5)	N7—C27—Cl2	113.9 (5)
C14—C13—C12	115.4 (5)	C27—C28—C29	119.2 (7)
C14—C13—H13A	122.3	C27—C28—H28A	120.4
C12—C13—H13A	122.3	C29—C28—H28A	120.4
N4—C14—C13	124.6 (6)	C28—C29—O12	121.7 (6)
N4—C14—Cl1	115.5 (5)	C28—C29—N8	124.1 (6)
C13—C14—Cl1	119.9 (5)	O12—C29—N8	114.2 (6)
O6—C15—H15A	109.5	O12—C30—H30A	109.5
O6—C15—H15B	109.5	O12—C30—H30B	109.5
H15A—C15—H15B	109.5	H30A—C30—H30B	109.5
O6—C15—H15C	109.5	O12—C30—H30C	109.5
H15A—C15—H15C	109.5	H30A—C30—H30C	109.5
H15B—C15—H15C	109.5	H30B—C30—H30C	109.5
			
O3—S1—N1—C10	176.9 (4)	O9—S2—N5—C25	−58.7 (5)
O4—S1—N1—C10	−54.2 (4)	O10—S2—N5—C25	173.5 (4)
C9—S1—N1—C10	61.1 (4)	C20—S2—N5—C25	57.8 (5)
C3—O1—C2—C1	−160.3 (6)	C18—O7—C17—C16	153.5 (6)
C2—O1—C3—O2	1.8 (9)	C17—O7—C18—O8	−3.6 (10)
C2—O1—C3—C4	−179.5 (5)	C17—O7—C18—C19	175.9 (5)
O2—C3—C4—C9	−46.4 (9)	O8—C18—C19—C24	125.1 (7)
O1—C3—C4—C9	135.0 (6)	O7—C18—C19—C24	−54.4 (7)
O2—C3—C4—C5	125.9 (7)	O8—C18—C19—C20	−51.7 (9)
O1—C3—C4—C5	−52.6 (7)	O7—C18—C19—C20	128.8 (6)
C9—C4—C5—C6	−2.5 (8)	C24—C19—C20—C21	−1.0 (8)
C3—C4—C5—C6	−175.4 (6)	C18—C19—C20—C21	175.7 (6)
C4—C5—C6—C7	4.8 (10)	C24—C19—C20—S2	176.5 (5)
C5—C6—C7—C8	−4.8 (10)	C18—C19—C20—S2	−6.7 (7)
C6—C7—C8—C9	2.5 (8)	O9—S2—C20—C21	7.4 (5)
C7—C8—C9—C4	−0.4 (8)	O10—S2—C20—C21	136.8 (5)
C7—C8—C9—S1	−175.3 (4)	N5—S2—C20—C21	−110.4 (5)
C3—C4—C9—C8	172.9 (5)	O9—S2—C20—C19	−170.2 (4)
C5—C4—C9—C8	0.4 (7)	O10—S2—C20—C19	−40.8 (5)
C3—C4—C9—S1	−12.5 (7)	N5—S2—C20—C19	72.1 (5)
C5—C4—C9—S1	175.0 (4)	C19—C20—C21—C22	−1.7 (9)
O3—S1—C9—C8	142.1 (4)	S2—C20—C21—C22	−179.3 (5)
O4—S1—C9—C8	10.0 (4)	C20—C21—C22—C23	2.6 (11)
N1—S1—C9—C8	−105.7 (4)	C21—C22—C23—C24	−0.8 (12)
O3—S1—C9—C4	−32.8 (5)	C20—C19—C24—C23	2.7 (9)
O4—S1—C9—C4	−164.9 (4)	C18—C19—C24—C23	−174.3 (6)
N1—S1—C9—C4	79.4 (4)	C22—C23—C24—C19	−1.8 (11)
S1—N1—C10—O5	−5.1 (7)	C26—N6—C25—O11	−177.9 (5)
S1—N1—C10—N2	176.5 (3)	C26—N6—C25—N5	2.3 (8)
C11—N2—C10—O5	179.5 (5)	S2—N5—C25—O11	10.6 (8)
C11—N2—C10—N1	−2.1 (7)	S2—N5—C25—N6	−169.5 (4)
C12—N3—C11—N4	−2.0 (7)	C27—N7—C26—N8	0.6 (8)
C12—N3—C11—N2	180.0 (4)	C27—N7—C26—N6	−176.7 (5)
C14—N4—C11—N3	2.1 (7)	C29—N8—C26—N7	0.9 (7)
C14—N4—C11—N2	−179.8 (4)	C29—N8—C26—N6	178.2 (4)
C10—N2—C11—N3	1.8 (7)	C25—N6—C26—N7	−6.2 (8)
C10—N2—C11—N4	−176.4 (4)	C25—N6—C26—N8	176.3 (5)
C11—N3—C12—O6	179.4 (4)	C26—N7—C27—C28	−1.1 (9)
C11—N3—C12—C13	0.0 (7)	C26—N7—C27—Cl2	177.4 (4)
C15—O6—C12—N3	−0.7 (8)	N7—C27—C28—C29	0.0 (11)
C15—O6—C12—C13	178.6 (6)	Cl2—C27—C28—C29	−178.3 (5)
N3—C12—C13—C14	1.6 (8)	C27—C28—C29—O12	−179.0 (6)
O6—C12—C13—C14	−177.8 (5)	C27—C28—C29—N8	1.7 (10)
C11—N4—C14—C13	−0.3 (8)	C30—O12—C29—C28	−180.0 (5)
C11—N4—C14—Cl1	178.8 (4)	C30—O12—C29—N8	−0.6 (7)
C12—C13—C14—N4	−1.5 (9)	C26—N8—C29—C28	−2.1 (8)
C12—C13—C14—Cl1	179.5 (4)	C26—N8—C29—O12	178.6 (4)

### Hydrogen-bond geometry (Å, °)

**Table:**

*D*—H···*A*	*D*—H	H···*A*	*D*···*A*	*D*—H···*A*
N1—H1A···N3	0.86	1.96	2.648 (6)	136
N2—H2A···O5^i^	0.86	2.00	2.831 (8)	162
N5—H5B···N7	0.86	1.96	2.651 (6)	136
N6—H6B···O2^ii^	0.86	2.14	2.973 (7)	162
C8—H8A···O4	0.93	2.43	2.821 (6)	105
C21—H21A···O9	0.93	2.44	2.848 (8)	107

Symmetry codes: (i) −*x*+1, −*y*+2, −*z*; (ii) *x*−1, *y*, *z*.
